# Macrophage efferocytosis in pregnancy and pregnancy complications

**DOI:** 10.1530/RAF-26-0041

**Published:** 2026-08-13

**Authors:** Chloe A Brady, Kylie B R Belchamber

**Affiliations:** Maternal and Fetal Health Research Centre, Division of Developmental Biology and Medicine, University of Manchester, Manchester, UK

**Keywords:** reproductive immunology, pregnancy, macrophage

## Abstract

**Abstract:**

Efferocytosis is a vital process during early placental development that removes apoptotic cells generated through decidualisation and controls inflammation. As pregnancy progresses and levels of apoptotic placental cells increase, macrophage efferocytosis remains essential to maintain a healthy pregnancy. There is mounting evidence that dysfunctional macrophage efferocytosis is implicated in the pathogenesis of multiple pregnancy complications. This includes disorders of malplacentation, such as preeclampsia and fetal growth restriction, wherein impaired spiral artery remodelling and inflammation in the decidua impair placental vascularisation leading to maternal hypertension, or impaired fetal growth. As a shift in macrophage phenotype and cytokine secretion is required for the onset of labour, alterations in phenotype earlier in pregnancy may be associated with preterm birth. In rarer pregnancy complications, such as chronic histiocytic intervillositis, impaired efferocytosis and wound healing by infiltrating intervillous, macrophages may contribute to their excessive recruitment into the intervillous space, hindering placental function. In this review, we describe the importance of efferocytosis by various maternal macrophage populations in the placenta and review the evidence of how impaired efferocytosis contributes to pathology. Furthermore, therapeutic targeting of macrophage efferocytosis and how this may be used to prevent or treat obstetric conditions are explored.

**Lay summary:**

During pregnancy, the body must safely clear away any dead cells in the womb to allow the placenta to grow and develop properly. This clean-up process is called efferocytosis, and it is carried out by immune cells known as macrophages. By removing these dying cells, macrophages help prevent inflammation and create the right environment for a healthy pregnancy. When efferocytosis does not work as it should, it can contribute to pregnancy complications. For example, if dead cells are not removed, the mother may develop high blood pressure, or the baby may not grow as expected. In rare pregnancy conditions, too many macrophages build up in the placenta. This build-up can interfere with the placenta’s ability to support the baby and can lead to stillbirth. In this review, we discuss why macrophage efferocytosis is so important for normal placental development and explore how problems in this process may lead to disease. We also consider new treatment ideas that could help control how macrophages work to prevent or manage pregnancy complications.

## Introduction

The uptake of apoptotic cells by macrophages, termed efferocytosis, meaning ‘to take to the grave’, is an essential process for tissue homeostasis and cellular turnover, as well as the resolution of inflammation (Doran *et al.* 2020). Rapid clearance of dying cells prevents leakage of potentially antigenic or cytotoxic contents into the extracellular milieu, which can initiate inflammation or tissue injury. Defects or delays in efferocytosis are associated with multiple pathologies, including cancer, autoimmune and inflammatory diseases (Vandivier *et al.* 2006). While macrophages are the main effector cells responsible for efferocytosis and appear to be fast eaters, other cells, including dendritic cells, and non-professional phagocytes, such as fibroblasts and epithelial cells, can also efferocytose, which eat more slowly and appear more selective about their prey (Sihombing *et al.* 2021).

In the context of pregnancy, efferocytosis holds particular importance due to the unique immunological and physiological demands at the maternal–fetal interface. In the developing placenta, levels of apoptosis are high due to rapid growth and remodelling of the uterus and local vasculature, including during spiral artery remodelling and extravillous trophoblast invasion into the decidua (Huppertz *et al.* 2006). Removal of these apoptotic cells by efferocytosis must, therefore, be tightly controlled to avoid excessive inflammation, which could result in the development of pregnancy complications. Preeclampsia, fetal growth restriction and preterm birth share common features of excessive inflammation and malplacentation, processes that may be exacerbated by inadequate efferocytosis. Understanding how this process occurs, how it is regulated and how it becomes dysregulated in disease may reveal novel insights into these complications and identify novel targets for therapeutic intervention.

## Efferocytosis

The process of macrophage efferocytosis is composed of four main steps (Moon *et al.* 2023). The first stage is recruitment: apoptotic cells release ‘find me’ signals such as sphingosine, adenosine and uridine triphosphate (ATP/UTP) and fractalkine, which act as chemoattractants for macrophages. The second stage is recognition, whereby apoptotic cells express ‘eat me’ receptors on their cell surface, which signal to the macrophage that the cell is undergoing apoptosis. These ‘eat me’ signals include phosphatidylserine (PS) and intercellular adhesion molecule (ICAM)-3, which bind to a variety of receptors on macrophages to initiate the internalisation process. ‘Don’t eat me’ signals, which prevent efferocytosis, include CD31, CD47 and CD24, which are reduced on apoptotic cells, but expressed on healthy cells (Ge *et al.* 2022). The third step is internalisation, in which ligand–receptor interactions activate downstream pathways to induce cytoskeletal rearrangement to facilitate ingestion of the large apoptotic cell. This includes recruitment of Rho GTPases and Rab molecules to aid movement of prey within the cell. Finally, digestion occurs, wherein ingested cells within a lysosome fuse with a phagosome for degradation by digestive enzymes. For a comprehensive review of these processes, see Moon *et al.* (2023) (Moon *et al.* 2023).

## Macrophages at the maternal–fetal interface

**Figure 1:**
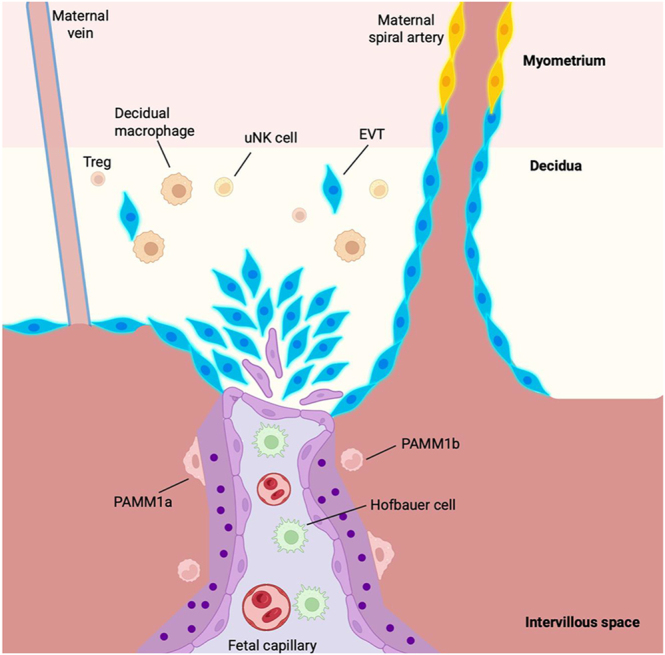
Macrophage populations in the placenta. There are three populations in the placenta. 1) Decidual macrophages – maternal-derived macrophages that populate the decidua and are important for spiral artery remodelling, tolerance generation and maintenance of an anti-inflammatory environment. 2) Placenta-associated maternal monocytes/macrophages (PAMMs) – maternal-derived monocytes (PAMM1b) and macrophages (PAMM1a) that populate the intervillous space where they are thought to contribute towards repair of the syncytiotrophoblast layer. 3) Hofbauer cells – fetal-derived macrophages that populate the villous stroma and maintain an anti-inflammatory environment in the fetal-side of the placenta. Created in BioRender, (https://BioRender.com/5k3pox5).

There are three main types of macrophage in the placenta: decidual macrophages, maternal intervillous macrophages and fetal-derived Hofbauer cells, residing within the placental villi (Fig. 1) (Bulmer & Johnson 1984). Decidual macrophages, which have recently been termed placenta-associated maternal monocyte/macrophage (PAMM)2, constitute around 20–30% of leukocytes at the maternal–fetal interface at implantation and remain high throughout pregnancy. The unique situation of this site, compared with other tissues, is the need for tolerance to be generated against the semi-allogenic fetus, to prevent rejection and promote a healthy pregnancy (Sun *et al.* 2021). This process is highly complex, as the phenotype of the maternal–fetal interface is not static. A pro-inflammatory environment is necessary for embryo implantation and placentation, which switches to a more tolerogenic phenotype required for fetal growth and development. Decidual macrophages must therefore display plasticity, altering in response to environmental cues and regulating the events of implantation (Rozner *et al.* 2016), placentation, vascular remodelling (Lash *et al.* 2016), fetal development and parturition (Vento-Tormo *et al.* 2018, Vondra *et al.* 2023). Maternal intervillous macrophages, also known as PAMM1a and PAMM1b, have recently been identified in the first-trimester placenta (Thomas *et al.* 2020) and appear to have important roles in repair of the syncytiotrophoblast (STB) layer and trophoblast differentiation (Thomas *et al.* 2021). Finally, Hofbauer cells, or fetal-derived macrophages, are found within the fetal villi and have important roles within the fetal side of the placenta (Reyes & Golos 2018). This review will focus mainly on maternal macrophages in the placenta.

### Macrophage phenotype

Macrophages are highly plastic and can switch phenotype and function depending on the needs of the local microenvironment. Traditional nomenclature places macrophages into M1 pro-inflammatory cells and M2 anti-inflammatory cells, which oversimplifies the high level of plasticity in response to stimuli and distinct locational phenotypes that have been observed (Lavin *et al.* 2014). Understanding the phenotypes and function of macrophages in the placenta is a complex and relatively young field, hampered by limited access to human samples and species differences with mice and other pregnancy-related animal models (Mestas & Hughes 2004). We must take care, therefore, in our discussion of pregnancy-related data.

The ontogeny of decidual macrophages, for example, has been established in mice but not humans. In mice, decidual macrophages appear to be composed of both maternal and embryonic macrophages, as well as bone marrow-derived macrophages, stimulated to proliferate by colony-stimulating factor-1 (CSF-1) in the myometrium (Tagliani *et al.* 2011). In humans, macrophages are found throughout the endometrium during all phases of the menstrual cycle, and are most abundant prior to menstruation. This rise in number suggests either *in situ* proliferation of endometrial macrophages or recruitment from blood monocytes; however, the evidence to support either theory is yet to exist (Brown *et al.* 2022). Decidual macrophages likely originate from endometrial macrophages at least at the start of pregnancy. In the first trimester, the decidua is enriched with monocytes which might suggest they differentiate into macrophages (Smárason *et al.* 1986, Vento-Tormo *et al.* 2018). This is likely a source of macrophages found in the intervillous space, the function of which is less understood (Thomas *et al.* 2021). However, as decidual macrophages can be identified in the first-trimester placenta prior to blood filling, they likely also originate from other sources, which could be endometrial macrophages or stem cells. Further research is needed to fully understand the ontogeny of all macrophage populations in the placenta across gestation.

### Efferocytosis during implantation

**Figure 2:**
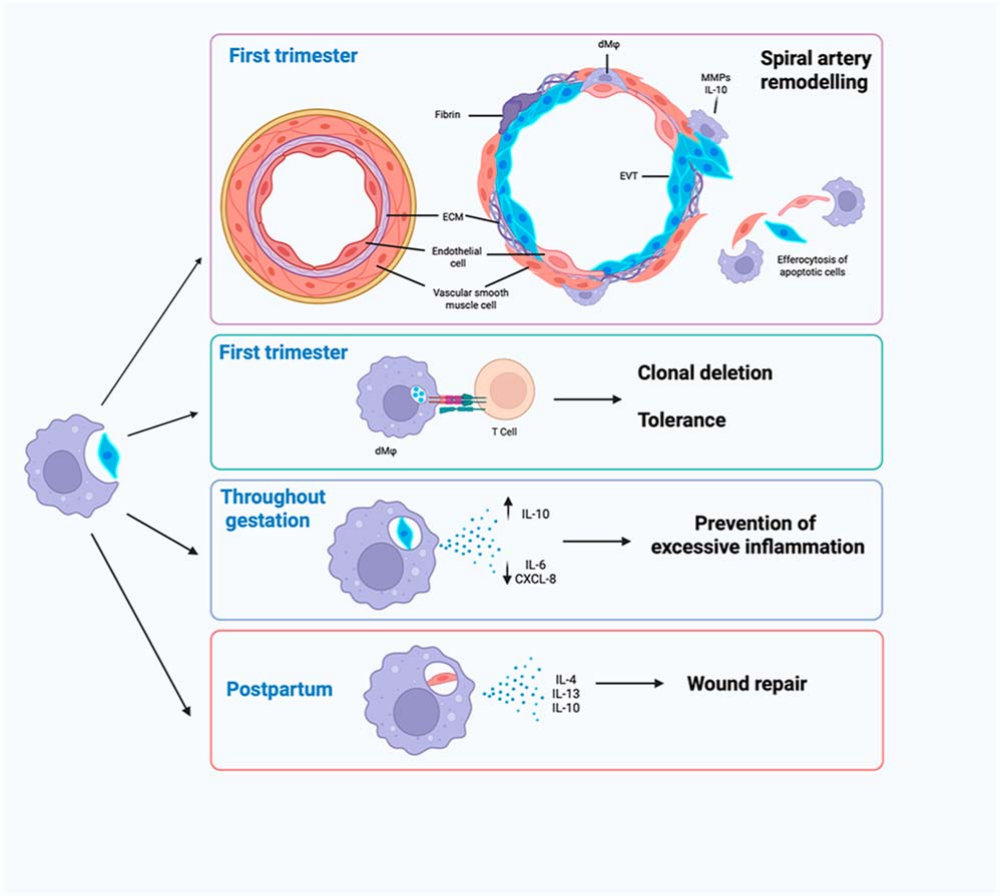
Role of macrophage efferocytosis in the placenta. Macrophage efferocytosis is important across gestation. In early pregnancy, decidual macrophages efferocytose apoptotic extravillous trophoblasts (EVTs), vascular smooth muscle cells and endothelial cells during spiral artery remodelling, to allow EVTs to infiltrate the vessel and widen spiral arteries. During this process, they secrete matrix metalloproteinases (MMPs), which degrade the extracellular matrix (ECM), as well as IL-10 to prevent excessive inflammation. Efferocytosis of EVTs and other fetal-derived placental cells results in antigen presentation to T cells, causing clonal deletion of T cells against fetal antigen, ultimately preventing rejection of the semi-allogenic fetus. Efferocytosis of EVTs and fetal-derived cells increases the release of anti-inflammatory IL-10 and inhibits the release of pro-inflammatory CXCL-8 and IL-6, preventing excessive inflammation throughout gestation. Postpartum, efferocytosis in the placental bed promotes wound healing and repair via secretion of IL-4, IL-13 and IL-10 (Kim & Nair 2019). Created in BioRender, (https://BioRender.com/haiz7bo).

For successful implantation, remodelling of the maternal endometrium must occur to allow invasion of the developing embryo and development of the decidua (Uckan *et al.* 1997). This remodelling induces a significant level of apoptosis within both maternal decidual cells and embryonic cells. Extravillous trophoblasts (EVTs), which express fetal MHC antigens (Holland *et al.* 2012), invade the myometrium during implantation where they plug spiral arteries while remodelling takes place. During this process, EVTs undergo apoptosis and are efferocytosed by decidual macrophages or dendritic cells, resulting in the expression of fetal antigen on the cell surface, which are then presented to T cells during normal antigen presentation (Faas *et al.* 2014). Elsewhere in the body, cross-presentation of self-antigens by dendritic cells leads to abortive proliferation of CD8^+^ T cells against self-antigens (Ma *et al.* 2024), important to avoid activation of an autoimmune response. However, in the placenta, this mechanism is likely important to avoid rejection of the semi-allogenic fetus. In mice, maternal antigen-presenting cells have been shown to take up apoptotic debris and present fetal antigens directly to T cells. CD8^+^ T cells that indirectly recognise the fetal antigen undergo clonal deletion without priming for cytotoxic effector function resulting in T cell ignorance of the fetus (Erlebacher *et al.* 2007) (Fig. 2). While these cell populations include all antigen-presenting cell populations, macrophages are capable of antigen cross-presentation (Muntjewerff *et al.* 2020), making this phenomenon likely to be shared across cells in the decidua. EVT apoptosis and efferocytosis during implantation is, therefore, necessary to generate maternal–fetal tolerance, and any impairment of this process may be implicated in pregnancy loss.

In humans, studies are beginning to emerge that support this theory. Monocyte-derived macrophage (MDM) efferocytosis of trophoblast debris has been shown to induce an anti-inflammatory phenotype including downregulation of co-stimulatory molecules CD80, CD86 and CD40, and upregulation of interleukin (IL)-10 secretion, driving a tolerogenic phenotype (Abumaree *et al.* 2012). Lipopolysaccharide-stimulated MDM can inhibit trophoblast invasion *in vitro*, supporting the theory that the correct inflammatory state of the decidua is required for successful implantation (Renaud *et al.* 2005, Renaud *et al.* 2007). Indeed, the removal of EVTs by efferocytosis prevents these trophoblasts from undergoing secondary necrosis, releasing pro-inflammatory intracellular components which would initiate inflammation. This is of utmost importance at the site of implantation, where too much inflammation can promote the rejection of the fetus. Murine uterine depletion of macrophages supports this idea, resulting in embryo implantation arrest, diminished plasma progesterone and poor uterine receptivity. This was alleviated with administration of macrophages (Care *et al.* 2013), indicating that these cells are vital for successful implantation.

### Spiral artery remodelling

During spiral artery remodelling, vascular smooth muscle cells (VSMCs) appear to be disrupted by the infiltration of macrophages and NK cells during early remodelling, but these immune cells are absent as remodelling finalises (Smith *et al.* 2009). Infiltrating macrophages and NK cells release matrix metalloproteinases (MMP)-7 and -9 to break down extracellular matrix and aid infiltration, which in turn increases apoptosis of VSMCs during remodelling (Lash *et al.* 2016). It is hypothesised that infiltrating decidual macrophages will efferocytose these apoptotic VSMCs, thus controlling the microenvironment and allowing remodelling to occur without excessive inflammation (Fig. 2). This has been demonstrated *ex vivo* but only occurred after 72 h of co-culture, which lags behind efferocytosis studies in other tissues and requires further validation (Lash *et al.* 2016).

### First trimester

#### Decidua

In the first trimester, extensive studies have explored decidual macrophage phenotype by a variety of methods. Single-cell sequencing studies have primarily targeted first-trimester human tissue. The Vento-Tormo *et al.* (2018) dataset identified two populations of decidual macrophages, termed dM1 and dM2, which express CSFR1 (the receptor for CSF1), as well as IL-10 (Vento-Tormo *et al.* 2018). However, further analysis of this dataset for decidual macrophage genes is yet to be carried out. By flow cytometry, three subsets of decidual macrophages have been identified based on CD11c and CCR2 expression, with 80% of cells being CCR2^−^ CD11c^low^ HMOX1*^−^*, located across the decidua and expressing anti-inflammatory genes. About 5% of macrophages were CCR2^−^ CD11c^high^, upregulating genes related to haem metabolism and cell proliferation, and were located close to EVTs in the decidua. Finally, 10–15% of macrophages were CCR2^+^ CD11c^high^, more pro-inflammatory by gene expression and similarly located in close proximity to EVTs. All macrophage subtypes were capable of bacterial phagocytosis, with the CCR2^−^ CD11c^high^ population most highly phagocytic *ex vivo* and had varying but overlapping transcriptional profiles. Authors proposed that these macrophages may have different functions, with CCR2^−^ CD11c^low^ involved in antigen presentation and anti-inflammation, CCR2^−^ CD11c^high^ primed for invading pathogen responses, and CCR2^+^ CD11c^high^ representing pro-inflammatory macrophages (Jiang *et al.* 2018).

A third study suggests two decidual macrophage populations separated as CD11c^high^ and CD11c^low^ populations. CD11c^high^ decidual macrophages expressed more lipid metabolism and inflammatory genes, and CD11c^low^ decidual macrophages expressed genes associated with extracellular matrix formation, muscle regulation and tissue growth. Both subsets secreted pro- and anti-inflammatory cytokines and could phagocytose plastic beads, although CD11c^high^ macrophages were more phagocytic (Houser *et al.* 2011). This matches the previous study, with CD11c^high^ macrophages identified as highly phagocytic which likely implicates them in efferocytosis, although this has not been measured in either study.

More generally, first-trimester decidual macrophages have been shown to release pro-inflammatory cytokines such as IL-6 and TNFα and anti-inflammatory cytokines such as IFNγ, IL-4 and IL-10 (Lidström *et al.* 2003, Svensson *et al.* 2011). Decidual macrophages also release IL-33, which induces proliferation of primary trophoblasts and cytotrophoblasts in explant cultures, suggesting that decidual macrophages can directly drive placental growth (Fock *et al.* 2013). In the first-trimester decidua, the existence of multiple macrophage phenotypes may be important to target inflammation to specific locations (such as spiral arteries) to facilitate specific functions, while limiting inflammation across other areas of the placenta. Whether these macrophage populations are consistently distinct, or if macrophages change phenotype based on specific local instructions remains to be determined. Plasticity in this context could be important, and lack of plasticity may contribute to impaired early placental development, as will be discussed later.

#### Intervillous space

From the seventh week of gestation, plugs of EVT within spiral arteries limiting blood flow into the placenta begin to degrade, and by the tenth week maternal perfusion of the intervillous space is fully established (James *et al.* 2017). Consequently, circulating maternal immune cells are brought into direct contact with fetal STB, representing the second site of the maternal–fetal interface after the decidua. Compared to those of the decidua, knowledge surrounding intervillous maternal monocytes and macrophages is limited, with most studies focusing on disease rather than the healthy placenta (Yankello *et al.* 2024). However, two single cell transcriptomic studies have identified specialised populations PAMM1s constituting 20–40% of isolated CD14^+^ cells during the first trimester (Vento-Tormo *et al.* 2018, Thomas *et al.* 2020). Thomas *et al.* (2020) describe PAMM1s as HLA-DR^high/low^FOLR2^−^ cells, differing from HLA-DR^high^FOLR2^high^ PAMM2s which are suggested to represent contaminating decidual macrophages. PAMM1 cells are further divided into PAMM1a and PAMM1b: PAMM1a exist as macrophages specific to the placental niche, while PAMM1b are monocytes transcriptionally similar to peripheral monocytes. PAMM1a may originate from peripheral monocytes or those migrating from the decidua, as placental villi have been shown to secrete cytokines and chemokines, including macrophage migration inhibitory factor (MIF) which can act as a chemoattractant (Solders *et al.* 2019, Thomas *et al.* 2020).

Of the PAMM population identified within the first-trimester placenta, PAMM1a cells are the most abundant (11% of CD14^+^ cells). Electron microscopy shows that PAMM1a can be found adhered to the STB in areas of damage, and it has been proposed that they are incorporated into the syncytium (Thomas *et al.* 2020). PAMM1a also exhibit high levels of intracellular lipid droplets which can be indicative of efferocytosis suggesting these cells may play a role in responding to damage and cell turnover at the maternal–fetal interface (Ford *et al.* 2019). A recent *in vitro* study showed that conditioned media from human first-trimester cytotrophoblast-derived HTR-8/SVneo cells can enhance efferocytosis in macrophages differentiated from peripheral blood monocytes, suggesting that the placenta can influence macrophage behaviour through soluble factors (Merech *et al.* 2026). Despite this, more extensive exploration of PAMM1a behaviour specifically is yet to be undertaken.

### Second trimester

As pregnancy progresses, decidual macrophages must maintain tolerance to the growing fetus. In the second trimester, decidual macrophages express high levels of CD86, CD163 and CD206, and secrete high IL-10 and transforming growth factor-β indicating an anti-inflammatory phenotype (Kwan *et al.* 2014, Wang *et al.* 2016); however, details on second trimester macrophages are limited by the availability of tissue for study at this point in gestation (Fig. 2).

### Third trimester

#### Decidua

By the third trimester, decidual macrophage expression of CD86, CD163 and CD206 drops, but they retain their ability to secrete anti-inflammatory cytokines and stimulate T cell proliferation (Heikkinen *et al.* 2003, Repnik *et al.* 2008, Wang *et al.* 2016). Two subsets of decidual macrophages, also characterised by CD11c expression, have been identified at term (Sureshchandra *et al.* 2023). CD11c^low^ macrophages express higher levels of alarmins and *CXCL-8*, whereas CD11c^high^ macrophages expressed higher levels of haemostatic genes including *TREM2* and *CD163*. How these cells differ in terms of function remains unassessed.

Higher rates of apoptotic cells have been identified in the third-trimester placenta compared to the first trimester, suggesting increased levels of prey for decidual macrophages (Smith *et al.* 2009). Efferocytosis, therefore, likely remains an important process throughout gestation; however, the evidence to support this remains limited. THP-1 macrophage-like cell lines have been shown to take up trophoblasts *in vitro* (Abrahams *et al.* 2004), with the rate of THP-1 efferocytosis positively correlating with trophoblast cell number. This suggests that as pregnancy progresses, macrophages may be able to respond to higher levels of apoptotic cells, important to maintain tolerance. We have recently shown that both decidua basalis and decidua parietalis macrophages isolated from uncomplicated, full-term placenta in the absence of labour are capable of efferocytosis of apoptotic trophoblasts, which inhibits release of pro-inflammatory CXCL-8 and IL-6 and stimulates the release of anti-inflammatory IL-10 (Belchamber *et al.* 2026*a*). We propose that this maintains a tolerogenic environment in the decidua throughout pregnancy, especially in the third trimester before the onset of labour. Further understanding of how this process occurs, and the effects of efferocytosis of VSMC and other key cell types will advance our knowledge of this area and highlight its importance throughout pregnancy.

#### Intervillous space

In the intervillous space of the third trimester placenta, the frequency of PAMMs is increased; PAMM1a and PAMM1b compose 8 and 21% of the immune cell population versus 3 and 13% in the first trimester, respectively. In contrast, the proportion of Hofbauer cells decreases from 38 to 4%. PAMMs expressed a range of immune surveillance and regulation receptors (CD62L, CD86, CD163) and were able to phagocytose *E. coli* beads, with PAMM1b the most highly phagocytic. Authors suggest these two cell types may have different roles based on transcriptomic profiles, which may be important for antimicrobial responses during pregnancy (Doratt *et al.* 2025). Further analysis of intervillous blood reveals that PAMM1b are phenotypically different to peripheral blood monocytes, with lower CD9 expression which may indicate a shift towards a more pro-inflammatory monocyte phenotype. Overall, this suggests that intervillous blood represents a unique immunological niche with regard to monocyte and macrophage phenotype and function, which requires further characterisation to understand these populations and their contribution to maternal–fetal tolerance.

### Labour

#### Decidua

The initiation of labour requires an inflammatory shift that occurs at the correct timing at the end of gestation. Single-cell sequencing studies of uterine tissue from term pregnancies show that monocytes and macrophages exhibit the most marked differences in response to labour, producing and responding to pro-inflammatory cytokines including IL-1β, IL-6 and IL-8 and promoting myometrial contractility (Pique-Regi *et al.* 2022). Decidual macrophage numbers increase during term and preterm labour (Hamilton *et al.* 2012), compared to non-laboured caesarean section deliveries. In the laboured placenta, higher levels of CD80^+^ macrophages were found, but no differences in levels of CD163^+^ CD209^+^ macrophages were found compared with non-laboured placentas, in both the decidua basalis and decidua parietalis (Xu *et al.* 2016). Authors suggest that this indicates an increase of M1-like macrophages; however, this cannot be determined through the expression of single markers. Decidual macrophages elevate expression of pro-inflammatory cytokines including IL-1β, IL-6 and CXCL-8 during labour which may trigger immune cell recruitment and activation of other cell types, and produce MMPs which may degrade fetal membranes (for review, see Paterson *et al.* (2025)). It is clear that decidual macrophages shift to a pro-inflammatory state to assist in the process of labour, but their true function during this process remains to be studied.

#### Intervillous space

In the intervillous space, labour causes mobilisation of monocytes into the placenta, specifically CD14^+^ CD16^+^ intermediate monocytes, as well as upregulation of CCL2 and M-CSF expression, markedly increased compared to peripheral blood sampled at the same time point (Vikberg *et al.* 2023). The intervillous space may, therefore, offer a bridging site between the placenta and the periphery, contributing towards inflammation required for spontaneous labour. Further work is required to understand how this links with decidual and placental inflammation, at which point it is likely that efferocytosis is no longer required as macrophages shift towards the pro-inflammatory phenotype required for labour.

### Postpartum

After delivery and placental detachment, the uterine wound left behind requires extensive repair during the postpartum period. This involves both innate and adaptive immune responses that must prevent infection and repair the endometrium. In mice, the postpartum wound is infiltrated with large numbers of macrophages and neutrophils on day 1, which resolves rapidly leaving a postpartum nodule which remains for 3 months and stains positive for macrophages throughout (Brandon 1994). A more recent study has shown that within the first 28 days of the postpartum period, the population of macrophages within the murine uterus is composed of a range of phenotypes including CD206^+^ CD86^+^ macrophages, as well as single positive populations, and levels of each vary throughout the repair process (Rivera *et al.* 2025). While this study assigned M1/M2 nomenclature to these cells, we know that this simplicity does not represent the true phenotype of macrophages. However, these data highlight that macrophage phenotype must shift as repair occurs. Depletion of macrophages using anti-F4/80 antibody created an increased area of senescent cells in the postpartum murine uterus, suggesting that macrophages are important for efferocytic removal of these cells (Egashira *et al.* 2017). Human studies are missing from this literature, likely due to the difficulty in studying the human postpartum uterus, but we postulate that macrophage efferocytosis is key during this process (Fig. 2). Failure in healing this wound could contribute to complications include postpartum haemorrhage, and the development of future pregnancy complications. In future, studies utilising lochia sampling may shed light on the function of macrophages within this crucial period.

## Efferocytosis in pregnancy complications

Pregnancy complications include conditions such as preeclampsia, intrauterine growth restriction (IUGR), preterm birth and stillbirth. While the pathophysiology of these conditions is diverse and complex, we will attempt to justify the potential role of macrophage efferocytosis. Defects in trophoblast invasion are associated with poor pregnancy outcome, with both reduced and excessive invasion contributing to pathology.

Reduced trophoblast invasion and failure to remodel spiral arteries is observed in preeclampsia and IUGR, whereas excessive trophoblast invasion is linked to placenta accreta and percreta, which cause postpartum haemorrhage (Sharma *et al.* 2016). Tight control of trophoblast invasion is, therefore, crucial for the establishment and maintenance of a healthy pregnancy.

### Recurrent spontaneous abortion

Miscarriage – pregnancy loss before 24 weeks of gestation – affects around 15% of clinically recognised pregnancies and is the most common obstetric complication. About 60% of miscarriages are associated with chromosomal abnormalities, while others are associated with maternal conditions including antiphospholipid antibodies, thyroid autoantibodies and subclinical hypothyroidism (Devall & Coomarasamy 2020). In the remainder of cases, the cause of miscarriage is unknown. Recurrent spontaneous abortion (RSA), or recurrent miscarriage, describes women who have had two or more miscarriages (Dimitriadis *et al.* 2020), often with unknown cause. Although the pathophysiology of recurrent miscarriage is complex and varies between individuals, there are associations with efferocytosis that are worthwhile to discuss.

**Figure 3:**
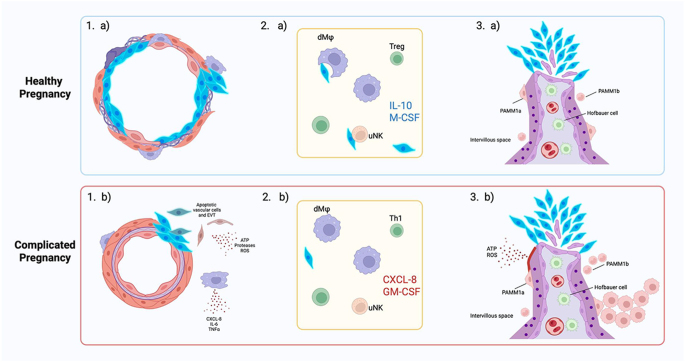
Dysfunctional efferocytosis in complicated pregnancy. 1) Spiral artery remodelling in healthy pregnancy (1a) vs defective spiral artery remodelling in complicated pregnancy (1b); impaired extravillous trophoblast (EVT) invasion of spiral arteries limits remodelling into the myometrium. During this process, EVTs, vascular smooth muscle cells and endothelial cells undergo apoptosis, failing removal by decidual macrophage (dMφ) efferocytosis, leading to secondary necrosis and the release of intracellular ATP, proteases and reactive oxygen species (ROS), driving inflammation in the placenta. 2) Anti-inflammatory environment in healthy pregnancy (2a) vs inflammatory environment in complicated pregnancy (2b); impaired dMφ efferocytosis drives a pro-inflammatory phenotype, which recruits Th1 cells over Tregs. The decidual environment becomes rich in pro-inflammatory cytokines, including CXCL-8 and GM-CSF, which drive inflammation. 3) Clear intervillous space during healthy pregnancy (3a) vs intervillous inflammation (3b); failure of PAMM1a function leads to impaired repair of syncytiotrophoblast (STB) damage, leading to the release of ATP and ROS. In chronic histiocytic intervillositis, this may lead to recruitment of large numbers of maternal macrophages, which invade the intervillous space and block placental function. Created in BioRender, (https://BioRender.com/).

Numerous studies have isolated decidual macrophages from placentas after RSA, showing that levels of macrophages are increased. These macrophages exhibit an inflammatory phenotype with higher levels of CD80 and CD86 and lower levels of peroxisome proliferator-activated receptors (PPAR)γ and IL-10 compared to controls (Jin *et al.* 2011, Wang *et al.* 2011, Kolben *et al.* 2018). This hints at an inflammatory macrophage phenotype, which could be targeted towards the semi-allogenic fetus and placenta, although this has not been proven (shown in Fig. 3(2b)). *In vitro*, low-molecular-weight heparin, often used to treat RSA, increased the expression of CD206, but not CD163 by decidual macrophages (Bruno *et al.* 2018), which again eludes to the idea that elevated inflammatory macrophage signalling, above that required in the first trimester, may be implicated in RSA.

Efferocytosis of decidual stromal cells by decidual macrophages has been shown to be elevated in RSA patients compared with controls (Sheng *et al.* 2018), alongside a disturbed IL-33/ST2 axis. The authors suggest two theories as to why this elevation in efferocytosis may occur. First, it may be a compensatory response to rescue the pregnancy and avert the release of autologous antigen. If this is insufficient, RSA may occur regardless. Alternatively, excessive efferocytosis may directly cause embryo loss, although the mechanisms of this are unclear. There appears to be a contradiction in the elevated pro-inflammatory, M1-like phenotype of RSA decidual macrophages and their apparent elevated efferocytic capacity, which would be expected to be decreased based on phenotype. Further functional and transcriptomic analysis of these populations is required to fully elucidate the true nature of decidual macrophages in cases of RSA, and uncover whether they are causative or activated in response to pathology.

One caveat of these data is that often tissue is collected from healthy control women recruited prior to undergoing elective medical or surgical termination of pregnancy. Comparatively, women experiencing RSA are recruited after medical diagnosis of no heartbeat, regardless of whether the tissue passed passively or surgically. Whether this affects the condition of the placenta and the activation of immune cells therein is unclear. Experiments where placentas following RSA are compared with those from singular miscarriage and medical terminations would be ideal to control for this, although this also has limitations.

### Early-onset preeclampsia

Early-onset preeclampsia (PE) is a condition of failed or inadequate placentation, characterised by impaired spiral artery remodelling resulting in hypoperfusion of the placenta, impaired EVT invasion and increased placental oxidative stress (Michalczyk *et al.* 2020). Initial studies of decidual macrophage phenotype in PE utilise immunohistochemical methods. These have shown that placental bed biopsies of PE pregnancies show increased numbers of decidual macrophages, but lower levels of trophoblast invasion compared to healthy pregnancies (Reister *et al.* 1999). Evidence of elevated decidual macrophage numbers in PE is conflicting; however, there is a consensus that these macrophages have increased pro-inflammatory M1-like phenotype, and decreased anti-inflammatory M2-like phenotype in PE (Wheeler *et al.* 2018). However, the data are muddied by the choice of markers. For example, Schonkeren *et al.* showed a decrease in CD14^+^ CD163^+^ ‘M2’ decidual macrophages by IHC in preterm PE placentas compared to non-PE preterm controls, but not term controls; however, the ‘healthy preterm control’ consisted of one quadruplet pregnancy, whose placentas were analysed individually to create four data points, which were then compared to singleton pregnancies, which complicates this analysis (Schonkeren *et al.* 2011). Overall, the classification of macrophage phenotype using one or two markers via immunohistochemistry is inferior compared with more advanced technologies which now allow more extensive characterisation. With this in mind, single-cell RNA sequencing datasets are beginning to emerge, providing support for these earlier reports. In PE, the CD11c^high^ decidual macrophage subset identified in healthy-term pregnancies had an activated transcriptomic signature with gene enrichment in chemotaxis, inflammatory responses and antigen presentation, which alludes to a role for efferocytosis. Spatial transcriptomics revealed CD11c^low^ macrophages had wide distribution, but CD11c^high^ macrophages were enriched around vascular endothelial cells indicating perivascular locations (Li *et al.* 2024). This supports the idea that decidual macrophages in PE are more inflammatory, and their location suggests that they might be involved in impaired spiral artery remodelling.

During spiral artery remodelling, decidual macrophages are thought to be key for the release of pro-angiogenic molecules, as well as MMPs to breakdown VSMC and allow EVT invasion (Helige *et al.* 2014). Disturbed inflammatory signalling by these macrophages might impair this process, leading to insufficient spiral artery remodelling and the pathogenesis of preeclampsia. This heightened inflammatory phenotype may also impair efferocytosis of apoptotic EVT and VSMC, further increasing inflammation. Our recent data show impaired efferocytosis of trophoblasts by decidual macrophages in placentas from early-onset PE compared with healthy controls, associated with elevated release of CXCL-8 and IL-6 (Belchamber *et al.* 2026*a*). The loss of ability to efferocytose at this location may cause these cells to die by secondary necrosis, which releases cell-free DNA and G-actin into the extracellular space (Fig. 3(1b)). This can enter the maternal blood and trigger damage to the maternal endothelium, contributing to the onset of hypertension (Huppertz *et al.* 2006). We postulate, therefore, that impaired decidual macrophage efferocytosis in PE may be causative of the disease; however, we cannot yet prove that this occurs early in pregnancy.

The aforementioned theory is supported by the observation of elevated levels of EVTs and cytotrophoblasts in the PE placenta (Crocker *et al.* 2003, Tomas *et al.* 2011, Petsas *et al.* 2012). Whether these are present due to placental damage or failed efferocytosis remains to be elucidated. *In vitro*, EVTs show increased rates of apoptosis when co-cultured with monocyte-derived macrophages and TNFα, suggesting this is a mechanism by which macrophages limit spiral artery invasion in the placental bed, but also that inflammatory cytokine secretion by decidual macrophages may promote elevated EVT apoptosis in PE (Reister *et al.* 2001). Serum levels of IL-1β, IL-6, IL-7, IL-8, IL-17a, MCP-1 and MIP-1β are elevated in PE compared to term controls, of which elevated IL-1β, IL-6 and MCP-1 can be detected within the placenta, indicating they may originate here (Ma *et al.* 2019).

Hofbauer cells in placentas from cases of early-onset PE also expressed higher levels of pro-inflammatory markers CD86 and HLA-DR, but lower CD209 compared to healthy control and late-onset PE, suggesting this inflammatory phenotype is not restricted to maternal immune cells. Uptake of zymosan (a yeast polysaccharide) was elevated only in Hofbauer cells from late-onset PE, which could be a consequence of this switch to a pro-inflammatory phenotype (Mercnik *et al.* 2023). Despite this, there is no evidence of altered efferocytosis by Hofbauer cells in PE or any pregnancy complication, representing an opportunity for future research.

Overall, it is clear that impaired macrophage efferocytosis is implicated in the pathogenesis of preeclampsia, resulting in increased levels of apoptotic cells in the placenta and a pro-inflammatory environment. This may impair spiral artery remodelling through insufficient removal of VSMC and endothelial cells, which then die by secondary necrosis and release mediators that damage maternal endothelium. This may contribute directly to the development of hypertension in the mother and impair vascular flow in the placenta. Targeting decidual macrophage function may, therefore, be a therapeutic target in women at risk of this complication.

### Preterm birth

Preterm birth is the spontaneous onset of labour before 37 weeks of gestation and is the leading cause of death in children under 5 years old (Ream & Lehwald 2018). Evidence of decidual macrophage involvement in preterm birth has been recently reviewed (Paterson *et al.* 2025), highlighting the lack of studies in this area. However, as decidual macrophages switch towards M1-like inflammation in both preterm and term labour (Xu *et al.* 2016), a premature switch in phenotype is the likely implication for decidual macrophage involvement in preterm birth. Whether they can be the cause of this event remains to be determined.

### IUGR/FGR

Fetal growth restriction (FGR), or intrauterine growth restriction (IUGR), is a condition where the fetus fails to reach its growth potential. While the causes appear to be due to abnormal placental development, FGR placentas also often contain lesions of maternal vascular malperfusion (MVM), fetal vascular malperfusion (FVM), and villitis of unknown aetiology (VUE) (Bezemer *et al.* 2024). Although MVM looks similar to the PE placenta regarding impaired spiral artery remodelling, increased trophoblast apoptosis and impaired EVT invasion, this may be less severe in FGR and limited to focal areas only. Placentas from cases of FGR can also be further complicated by increased fibrin deposition, as well as reduced villous volume and surface area for maternal–fetal exchange (Burton & Jauniaux 2018).

Impaired maternal–fetal tolerance has been proposed as one of the causes of FGR, with increased macrophages found in placentas of fetuses with a decreased growth rate compared to those small for gestational age, not defined as FGR (Sharps *et al.* 2020). These FGR placentas exhibit elevated IL-8 and elevated maternal uric acid in placental tissue, suggesting non-infectious inflammation. Further studies have confirmed elevated CD68+ decidual macrophages in FGR, with decreased CD206 expression highlighted via immunohistochemistry, which may indicate reduced anti-inflammatory macrophage populations (Bezemer *et al.* 2020). There also appear to be increased maternal macrophages in the intervillous space of FGR pregnancies, likely from maternal monocyte migration (Chu *et al.* 2019), which may contribute to inflammation in this condition. Overall, there is a lack of evidence of macrophage dysfunction in FGR, and this remains a condition that needs further analysis.

### Gestational diabetes mellitus

Gestational diabetes mellitus (GDM) occurs in around 14% of pregnancies and is defined as glucose intolerance first detected during pregnancy, which resolves after birth (Wang *et al.* 2022). The role of macrophages in GDM was recently reviewed elsewhere (Milan *et al.* 2025), and so we will focus on efferocytosis here. Hyperglycaemia induces inflammatory gene expression in macrophages which may persist even after a return to normal glucose levels (Edgar *et al.* 2021). This includes downregulation of the scavenger receptor CD163, important for haemoglobin clearance and phagocytosis (Matuschik *et al.* 2022). Overall, the shift to a pro-inflammatory macrophage phenotype, supported by generalised inflammation in the GDM placenta, may support an impairment of efferocytosis by decidual macrophages and Hofbauer cells, although there is currently a lack of evidence to support this hypothesis.

### Chronic histiocytic intervillositis

Chronic histiocytic intervillositis (CHI), also known as chronic intervillositis of unknown aetiology (CIUE), is a placental lesion where CD68^+^ maternal macrophages accumulate excessively in the intervillous space (Bos *et al.* 2018) (Fig. 3(3b)). CHI can occur in any trimester, affecting around 0.7% of pregnancies and is strongly associated with FGR, recurrent miscarriage and stillbirth (Simula *et al.* 2024). The live birth rate in affected pregnancies is reported around 53%, and there is no standardised treatment proven to be effective (Feist *et al.* 2023, Simula *et al.* 2024). Currently, the cause of CHI is unknown, although a predominating hypothesis is that it is a form of maternal immune rejection towards paternally inherited placental antigens. This is supported by case series wherein CHI manifests discordantly in placentas of dizygotic twin pregnancies, suggesting an alloimmune component. Multiple groups have explored the comparison between CHI and antibody-mediated allograft rejection, although a causal link with maternal anti-HLA antibodies is disputed as placental HLA expression is restricted, and sensitisation also occurs following a healthy pregnancy (Vilches & Nieto 2015, Brady *et al.* 2024).

Krop *et al.* found three distinct macrophage clusters of intervillous CD68^+^ cells in placentas with CHI expressing an array of different receptors, but their functions are elusive. These macrophages express scavenger receptors including CD204, CD163 and CD68 hinting at efferocytosis potential, but further analysis is required (Krop *et al.* 2023). New evidence from a study of first-trimester placentas with CHI also revealed that intervillous macrophages demonstrate various upregulated genes related to cholesterol and lipid metabolism, suggesting these as potential disease mechanisms related to efferocytosis (Kailasam *et al.* 2026). Areas of macrophage infiltration in CHI correlate with decreased STB levels of CD39, an enzyme which regulates extracellular ATP levels via conversion to adenosine monophosphate (AMP) (Krop *et al.* 2023). In a study of healthy placentas, CD39 expression was 2.3-fold higher at term compared to the first trimester, likely encouraging extracellular ATP clearance as trophoblast apoptosis increases with gestation (Forstner *et al.* 2023). Therefore, impaired CD39 expression in CHI may lead to elevated ATP levels in the intervillous space, acting as a DAMP to recruit monocytes and macrophages. The chemoattractant ICAM-1 has been found to be significantly elevated in STB and infiltrating maternal macrophages in placentas with CHI, which may explain increased immune cell aggregation in the intervillous space (Labarrere *et al.* 2014, 2015). These changes in immune-related proteins appear confined to the intervillous space, with no alterations in expression within villous or decidual tissue (Broekhuizen *et al.* 2021). This suggests a maternally driven pathology, the cause of which remains unknown.

As well as CD68^+^ macrophages, maternal T cells including regulatory T cells (Tregs) are also present within the intervillous infiltrate in CHI, albeit to a lesser degree (∼5% of mononuclear cells (Capuani *et al.* 2013, Labarrere *et al.* 2015)). This increase in maternal Treg and macrophage recruitment has led to the hypothesis that CHI represents an ineffective attempt to resolve placental inflammation (Terry 2024), but the pathways leading to increased immune recruitment are largely unknown. Historically, functional analysis of macrophage behaviour in CHI has been limited, as diagnosis can only be made retrospectively using formalin-fixed paraffin-embedded tissue. To address this, intervillous blood sampling of placentas from pregnancies at risk of recurrent CHI has been proposed, which hopes to allow real-time investigation of macrophage dynamics and shed light on pathophysiology (Lu *et al.* 2025).

## Therapeutic potential

**Figure 4:**
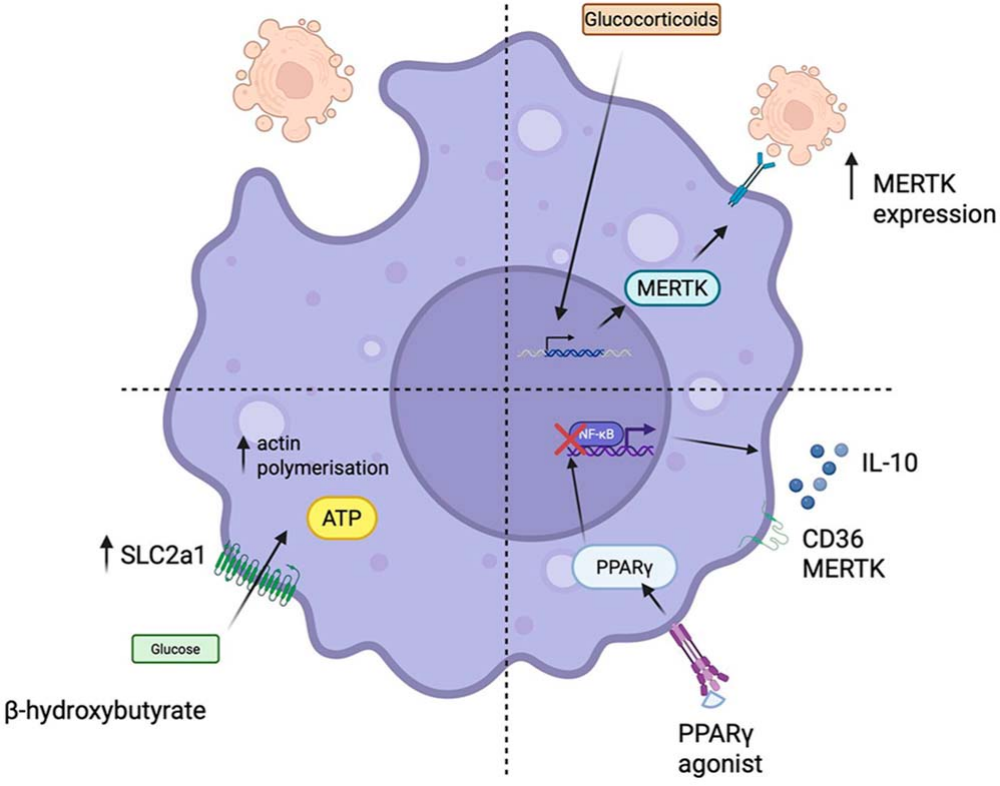
Therapeutic targets to elevate efferocytosis. Potential therapies that improve efferocytosis include PPARγ agonists (pioglitazone), which activate PPARγ, blocking NFκB transcription of pro-inflammatory genes, elevating expression of efferocytic receptors, including CD36 and MERTK, and elevating release of anti-inflammatory IL-10. Drugs such as β-hydroxybutyrate elevate glucose transporters, such as SLC2a1, which increase energy supply to the cell required for actin rearrangement during efferocytosis. This may increase the ability of the cell to actively efferocytose and breakdown cargo. Finally, glucocorticoids have been shown to activate transcription of efferocytic receptors, including MERTK, elevating efferocytosis in mice *ex vivo*. These potential therapies might prove beneficial in pregnancy, but require further testing. Created in BioRender, (https://BioRender.com/yuwqal9).

Macrophage efferocytic function is implicated in many pathologies outside of those affecting pregnancy, and efforts are underway to manipulate this process as potential therapy in cancer, wound healing and other inflammatory diseases (Mehrotra & Ravichandran 2022, Li *et al.* 2026). The obstetric field must, therefore, pay attention to these therapies to understand whether they might be utilised to prevent or treat pregnancy complications, without harming the developing fetus. Promising treatments include PPARγ agonists such as pioglitazone used in type 2 diabetes. PPARγ agonism can boost macrophage efferocytosis (Belchamber *et al.* 2026*b*) and induce anti-inflammatory gene expression through trans-repression of NFκB and other pro-inflammatory genes (Rigamonti *et al.* 2008, Fernandez-Boyanapalli *et al.* 2015), suggesting that it could improve macrophage function in disorders of malplacentation, including PE and FGR (Fig. 4). Treatment of *ex vivo* decidual macrophages from patients with preterm birth with rosiglitazone, another PPARγ agonist, reduced TNFα expression, suggesting a switch towards an anti-inflammatory phenotype, and was able to prevent LPS-induced preterm birth in mice (Xu *et al.* 2016). In obese mice, erythropoietin elevated PPARγ expression by macrophages to increase efferocytosis (Luo *et al.* 2019), further supporting this pathway as a potential target. Vasoactive intestinal peptide (VIP) has also been shown to increase implantation rates in mice, partly through elevated PPARγ expression in the placenta, and by elevating peritoneal macrophage efferocytosis (Gallino *et al.* 2016). These small studies point towards the PPARγ pathway having potential in both early and late pregnancy to stimulate efferocytosis leading to positive pregnancy outcomes.

Further potential therapies include β-hydroxybutyrate, a dietary supplement currently undergoing human clinical trials for treatment of type-1 diabetes. β-Hydroxybutyrate increases expression of solute carrier proteins including *SLC2a1* (GLUT1) (Yurdagul *et al.* 2020), which increases glucose uptake to the efferocytic cell. As efferocytosis is an energy demanding process, especially necessary for actin and dynein rearrangement, elevating glucose uptake has been shown to increase levels of efferocytosis experimentally (Morioka *et al.* 2018) (Fig. 4). In mice, glucocorticoids have been shown to rescue a drop in efferocytosis caused by lipopolysaccharide, via increased transcription of *MERTK* and other related genes, suggestive of an improvement in efferocytosis in cases of infection (Michlewska *et al.* 2009, Garabuczi *et al.* 2015). Many other therapies are currently being tested in cancer, atherosclerosis, wound healing and arthritis (Mehrotra & Ravichandran 2022, Gao *et al.* 2026). These therapies target various aspects of the efferocytic pathway, including elevating ‘eat me’ receptor targets on macrophages, such as TIM3 and 4 (Sheng *et al.* 2024). While these strategies may elevate efferocytosis in cancer-specific studies, their efficacy and safety in pregnancy require investigation. Before we can identify novel therapies safe for pregnancy, advancing our understanding of specific defects in macrophage efferocytosis within the placenta is vital.

## Limitations in the field

Compared to other areas of immunology, placental immunology remains in its infancy, and while huge strides have been taken in recent years to understand the role of the immune system in pregnancy, knowledge is still lacking. As a result, many assumptions about the roles of macrophages and efferocytosis in pregnancy have been made, but true functional evidence is required. Several murine studies have not been included in this review, due to interspecies differences in macrophage populations (Mestas & Hughes 2004), as well as placental structure and function (Schmidt *et al.* 2015). Studies utilising cell lines or monocyte-derived macrophages alone lack the input of the local microenvironment, which hugely influences macrophage phenotype and function (Lavin *et al.* 2014). Investigation of patient-derived macrophages and creation of complex models which consider the placental immune niche are, therefore, warranted to better understand the dynamic process of efferocytosis and how this may be aberrant in disease.

## Conclusion and future perspectives

Macrophage efferocytosis within the placenta contributes to early embryo implantation, spiral artery remodelling and the generation of tolerance to the fetus. As pregnancy progresses, efferocytosis remains important to maintain a homeostatic, anti-inflammatory environment to preserve the pregnancy and avoid excessive inflammation in the decidua and intervillous space. Any disturbance in this process may contribute to the onset of pathology and preterm labour. Despite this, understanding of macrophage efferocytosis within the placenta is limited, and it remains to be answered how the ingestion of large cargo may affect macrophage function and whether this is altered in pregnancy complications. Efferocytosis represents a potential therapeutic target in PE, FGR, CHI and other pregnancy complications worthy of ongoing study.

## Declaration of interest

The authors declare that there is no conflict of interest that could be perceived as prejudicing the impartiality of the work reported.

## Funding

This research did not receive any specific grant from any funding agency in the public, commercial or not-for-profit sector.

## Author contribution statement

KBRB conceived the review. CAB and KBRB drafted the article. Both authors approved the final version.
